# Supplementary material for: PARP Inhibitors in Pancreatic Cancer with Homologous Recombination Repair Gene Mutations: A Single-Institution Experience
Source: Cancers (Basel). 2024 Oct 11;16(20):3447. doi: 10.3390/cancers16203447 (PMC11505755; doi:10.3390/cancers16203447)
Supplement: Supplementary file 1 [file cancers-16-03447-s001.zip › cancers-3211805-supplementary.pdf]

Supplementary Data

Table S1. Mutated HRR genes.

Patient No.	Germline					Somatic				
	**Gene**	**Mutation**	**Zygosity**	**Assay**	**ClinVar ***	**Gene**	**Mutation**	**Variant Allele Frequency**	**Assay**	**ClinVar ***
1	*BRCA2* [#]	NA [#]	NA [#]	NA [#]						
2	*BRCA2*	*c.5946del*	Heterozygous	Invitae	P					
3	*BRCA2*	*c.5946del*	Heterozygous	Invitae	P	*ATM*	*R3008C*	0.6%	Guardant360	P/LP
4	*BRCA2*	*c.8488-1G>A*	Heterozygous	Invitae	P					
5	*BRCA1*	*187delAG*	NA	Myriad Genetics	P					
6	*BRCA2*	*c.5946del*	Heterozygous	Invitae	P					
7						*BRCA2*	*E2846fs *22*	NA	FoundationOne CDx	P
8	*CHEK2*	*c.1461+1G>A*	Mosaic	Invitae	P/LP					
9	*PALB2*	*Gain (Exon11)*	Copy number = 3	Invitae	LP					
10	*BARD1*	*Deletion (Exon1)*	Heterozygous	Invitae	P					
11	*ATM*	*c.8147T>C*	Heterozygous	Invitae	P/LP					
	ATM	*c.4668T>G*	Mosaic	Invitae	P					
12						*CHEK2*	*Q330 **	0.18%	FoundationOne Liquid CDx	P
13						*BAP1*	*M80Sfs *7*	8.8%	Moffitt Star	P
14	*RAD51C*	*c.145+1G>T*	NA		P/LP	*ARID1A*	*Q944fs *14*	NA	FoundationOne CDx	NA
15						*NBN*	*c.896+1G>A*	16%	CARIS	LP
						ARID1A	*p.L2089fs*	31%	CARIS	NA
16						*ATM*	*Splice site SNV*	49%	Guardant360	P/LP
						ATM	*R337C*	0.4%	Guardant360	VUS
						ATM	*G2891D*	0.1%	Guardant360	VUS
						ATM	*c.1791_1802+15del*	1.7%	Guardant360	NA
						ATM	*c.3567_3576+1del*	0.9%	Guardant360	NA
17	*ATM*	*c.3802del*	Heterozygous	Invitae	P					
18	*NBN*	*c.657_661del*	Heterozygous	Invitae	P	*BRCA2*	*K2729fs *3*	NA	FoundationOne CDx	P
19	*CHEK2*	*c.470T>C*	NA	Myriad Genetics	Conflicting, P/LP/VUS					
20	*WRN*	*Deletion (Exon 19-23)*	Heterozygous	Invitae	P					
21						*ARID1A*	*R1276 **	NA	FoundationOne CDx	P
							*Q2176 **	NA	FoundationOne CDx	NA
22						*ATM*	*c.7629+1G>A*	NA	FoundationOne CDx	P/LP
23	*BRCA2*	*c.4638del*	Heterozygous	Invitae	P					
24	*ATM*	c.3576G>A	Heterozygous	Invitae	P/LP	*ATM*	*S743 **	0.4%	Guardant360	P
25	*BRCA1*	*R1203**	Heterozygous	Ambry Genetics	P					
26	*PALB2*	*c.2325dup*	Heterozygous	Invitae	P	*BRCA2*	*S1632fs*	0.2%	Guardant360	P

27	*ATM*	*p.M1?*	NA	Ambry Genetics	P/LP					
	FANCC	*c.67delG*	NA	Ambry Genetics	P					
28	*FANCA*	*c.4285G>A*	Heterozygous	Invitae	Conflicting, LP/VUS					
29	*BRCA2*	*c.658_659del*	Heterozygous	Invitae	P					
	NBN	*c.657_661del*	Heterozygous	Invitae	P					
30						*ATM*	*R3008C*	0.5%	Guardant360	P/LP
31	*PALB2*	*c.3549C>G*	Heterozygous	Invitae	P	*PALB2*	*c.2559C>T*	19%	CARIS	LP
32	*RAD51C*	*c.224dup*	Heterozygous	Invitae	P					
	FANCA	*c.862G>T*	Heterozygous	Invitae	P					
33	*FANCA*	*c.4199G>A*	Heterozygous	Invitae	Conflicting, P/LP/VUS					
34						*ATM*	*C790 **	0.16%	FoundationOne CDx	NA
35						*BRCA2*	*c.755_758del*	NA	CARIS	P
36	*BRCA2*	*c.6275_6276del*	NA	Ambry Genetics	P					
37						*ATM*	*L1255fs*	0.8%	Guardant360	P/LP
38	*FANCM*	*c.1581+1G>A*	Heterozygous	Invitae	LP					
39	*ATM*	*c.8147T>C*	Heterozygous	Invitae	P/LP					
40	*FANCG*	*c.1795_1804del*	Heterozygous	Invitae	P					
41	*BRCA2*	*c.3847_3848del*	Heterozygous	Invitae	P					
	BRIP1	*c.751C>T*	Heterozygous	Invitae	P/LP					
42	*BRCA1*	*c.4035del*	Heterozygous	Invitae	P					
43	*FANCM*	*c.5101C>T*	Heterozygous	Invitae	P/LP					
44	*ATM*	*c.663-2A>G*	Heterozygous	Invitae	LP					
45	*BRCA2*	*c.4472_4475del*	Heterozygous	Myriad Genetics	P					
46	*BRCA2*	*c.5946del*	NA	Myriad Genetics	P					
47						*BRCA2*	*c.289G>T*	0.6%	Guardant360	P
48	*NBN*	*c.585-1_585delGTinsC*	NA	Ambry Genetics	LP					

* https://www.ncbi.nlm.nih.gov/clinvar/ (accessed on 29 September 2024). # Germline pathogenic BRCA2 mutation was documented in patient's chart; however, a genetic testing report was not available. NA, not available. P, pathogenic. LP, likely pathogenic. VUS, variant of uncertain significance.
